# Purification and characterization of a noble thermostable algal starch liquefying alpha-amylase from *Aeribacillus pallidus* BTPS-2 isolated from geothermal spring of Nepal

**DOI:** 10.1016/j.btre.2020.e00551

**Published:** 2020-11-01

**Authors:** Parash Mani Timilsina, Gyanu Raj Pandey, Asmita Shrestha, Manish Ojha, Tika Bahadur Karki

**Affiliations:** a Department of Biotechnology, Kathmandu University, Dhulikhel, 6250, Nepal; b Biotechnological Research and Developmental Center, Bharatpur, Chitwan, 44200, Nepal

**Keywords:** SDS-PAGE, Sodium dodecyl sulphate polyacrylamide gel electrophoresis, K_m_, Michaelis constant, DEAE-Cellulose, Diethylaminoethyl cellulose, TLC, Thin layer chromatography, Alpha-amylase, Enzyme kinetics, Thermostability, Microalgal biomass, Liquefaction, Viscosity, Starch

## Abstract

A thermophilic strain, *Aeribacillus pallidus* BTPS-2 was isolated from Bhurung geothermal spring of Nepal. The 16 s rRNA sequence showed 99.8 % similarity with the type strain *Aeribacillus pallidus* DSM 3670. The morphological, physiological and biochemical properties were similar to the type strain. Alpha-amylase from *A. pallidus* BTPS-2 was purified to 19-fold purification by DEAE-Cellulose ion exchange chromatography. The K_m_ value of amylase on starch was 0.51 ± 0.05 mg/mL. The optimum pH and temperature were 7.0 and 70 °C. SDS-PAGE analysis showed a single band at 100 kDa. The half-life of the enzyme at 80 °C was 2.81 h. The enzyme showed an inhibitory effect in the presence of Fe^2+^, Pb^2+^, Sn^2+^ and Hg^2+^ at 10 mM concentrations. TLC analysis showed that the enzyme is a liquifying alpha-amylase. The enzyme reduced the viscosity of algal biomass suspension up to 74.2 ± 0.17 % which was more efficient than *Bacillus amyloliquefaciens* alpha-amylase (80.5 ± 0.2 %).

## 1 Introduction

Bioethanol is commercially produced from the fermentation of food crops such as corn, sugarcane and beets [1]. Algal feedstock, however, is considered as the most promising non-food feedstock that can be used for biofuel production [2]. Recent studies suggested that microalgae can be a better candidate for ethanol production due to the absence of lignin, low cellulose, and high starch content [3]. Enzymatic pretreatment of algal biomass such as liquefaction and saccharification increases bioethanol yield by several folds [4]. Studies have been done for the identification of an efficient process for algal biomass pretreatment. For example, ethanolic fermentation by *Zymomonas mobilis* on amylase treated spirogyra increased bioethanol yield by several folds [5]. Similarly, the two-stage hydrolysis of *Graciliaria salicronia* resulted in a higher yield of glucose [6]. Enzymatic hydrolysis of *Chlorella vulgaris* biomass containing 51 % starch gave a glucose yields of 90.4 % [7]. Among all pre-treatments used, the enzymatic treatment with thermostable enzymes showed the highest ethanol recovery [8].

Thermostable alpha-amylase is active and stable at high temperatures, such as during gelatinization and liquefaction of starch. Thermostable enzymes are thus a potential candidate for industrial application in starch pre-treatment. A study on commercial enzymes for saccharification of algal biomass also showed promising results [9]. Enzymatic activity depends on various factors such as pH, temperature, salinity and presence of metal ions. Algal biomass is a complex mixture of organic and inorganic components that may affect the enzymatic starch hydrolysis process. Thus, a search for a novel enzyme that can effectively liquefy algal biomass is needed.

*A. pallidus* was first isolated by Scholz et al. from thermophilically treated wastewater [10]. The genus was changed to *Aeribacillus* from *Geobacillus* on account of its unique physiological and phylogenetic characteristics [11]. *A. pallidus* has been isolated from other diverse habitats such as high-temperature oil fields [12] and geothermal springs [[13], [14], [15]]. Some strains of *A. pallidus* are a potential source of industrially important enzymes such as protease [16,17] and lipase [18,19]. However, there has been no study available in the relevant literature for the purification and characterization of alpha-amylase from *A. pallidus*

In this study, a highly amylolytic *Aeribacillus pallidus* BTPS-2 strain was isolated from Bhurung geothermal spring sediment of Nepal. *A. pallidus* BTPS2 was characterized by morphological, physiological, biochemical and molecular techniques. Optimum media for amylase expression was selected. The enzyme was purified to homogeneity and characterized. The liquefaction efficiency of starch-rich algal biomass was compared with the alpha-amylase from *Bacillus amyloliquefaciens.*

## 2 Material and methods

### 2.1 Materials

All bacteriological grade growth medium and reagents were purchased from Himedia. Analytical reagents for enzyme assay, TLC, SDS-PAGE, buffers, *B. amyloliquefaciens* alpha amylase, dialysis membrane, and TLC Plates were purchased from Sigma-Aldrich. The starch quantification kit was purchased from Megazyme. The dry biomass of *Chlorella vulgaris* was obtained from the Algae Research Laboratory, Department of Biotechnology, Kathmandu University. All statistical analyses were done in Minitab 19.0.

### 2.2 Sample collection and isolation

Samples were collected from Bhurung geothermal springs located in Myagdi district of Nepal. Water, biomats and sediments were collected using three bottles of 500 mL sterile Thermo flask and transported to the laboratory within 24 h. The surface water temperature was recorded in situ. pH reading was taken at 25 °C.

For enrichment of thermophiles, 1 mL of the sample from water, biomats and sediment suspension was added to 10 mL nutrient broth (pH 7.0) and incubated in water bath at 55 °C. Growth was monitored by measuring turbidity with a DEN-B densitometer. Isolation of pure culture was done by spread plate and streak plate techniques on nutrient agar pH 7.0. Homogeneous colonies were transferred to sterile 5 mL nutrient broth in 15 mL culture tubes and incubated at 70 °C corresponding to the temperature of the sample site. Densitometric reading was done every 2 h to monitor the growth. The culture that showed positive growth was inoculated in nutrient broth at 37 °C. The culture that showed growth at 70 °C but does not show significant growth at 37 °C was screened for amylase activity. The alpha-amylase activity was tested by streaking the pure culture in 1 % Starch agar plates. The amylolytic activity was detected by the formation of a clear zone around the isolates after the addition of Lugol's iodine. The strain with a positive amylase test was subcultured in nutrient agar at 55 °C to obtain pure colonies. The strain was stored in nutrient broth with 20 % glycerol at −80 °C. Slant culture is also prepared for regular subculture and stored at 4 °C.

### 2.3 Characterization of the isolates

The strain with positive amylase activity was characterized by the phenotypic, physiological and molecular study. The phenotypic study investigated were colony morphology, grams staining, spore staining and microscopic observations. Colony morphology was done in nutrient agar plates after 48 h incubation at 55 °C. Grams staining and spore staining was done according to standard microbiological methods.

The physiological parameters studied were: optimum growth temperature, optimum pH, optimum salt tolerance and motility test. Optimum temperature, pH and salt concentration were obtained by OVAT (one variable at a time) method. For determination of optimum temperature, 0.1 mL overnight culture was transferred to 5 mL fresh nutrient broth in 15 mL tubes. The tubes were incubated at 40 °C, 50 °C, 60 °C, 70 °C and 80 °C in orbiter shaker at 150 rpm. The optical density readings were taken every 2 h. For determination of optimum pH, the temperature was maintained at previously identified temperature optimum and inoculated in nutrient broth with pH 6.0, 6.5, 7.0, 7.5, 8.0 and 8.5. For the determination of optimum salt tolerance, overnight log phase culture was transferred to 5 mL nutrient broth with final NaCl concentrations of 0 %, 1 %, 2 %, 3 %, 4 % and 5 %. Suitable dilution was done to maintain initial OD at 0.1 and incubated in an orbital shaker. OD reading was done in 2 h interval. The maximum specific growth rate was estimated by the Gompertz growth model using the nonlinear regression method [20]. The motility test was studied by stab culture in SIM medium and observation of the culture diffusion after 24 h incubation at optimum temperature.

Physiological characteristics such as VP test, phenylalanine deaminase test, anaerobic growth, nitrate reduction test, oxidase test catalase test was done by standard microbiological methods. Casein hydrolysis, Gelatin hydrolysis, starch hydrolysis and urea decomposition test were done according to Scholz et al. [10]. All carbohydrate utilization test was done in replicates using the Himedia HiCarbo™ identification kit. Antibiotic sensitivity test for 16 different antibiotics was done with the Himedia HiComb™ disc according to the manual.

### 2.4 Molecular characterization

Genomic DNA was isolated using DNeasy Ultraclean Microbial Kit Qiagen. PCR amplification was performed using 27 F and 1492R universal primers. The PCR product was purified by the Qiagen QIAquick PCR purification kit. The PCR product was sequenced in ABI Prism DNA sequencer (Macrogen, Korea) using Big Dye terminator cycle sequencing. Sequencing primers used were 785 F and 907R primers. The consensus sequence was generated in Mega-X. The FASTA sequence was analyzed in the NCBI blast tool. The phylogenetic tree was constructed using the neighbor Joining algorithm. The sequence was published in NCBI GenBank.

### 2.5 Determination of amylase assay

The activity of alpha-amylase was determined according to Bernfeld et al. [21]. The unit of alpha-amylase is defined as the amount of enzyme which liberates 1 mg of reducing sugar as maltose in 3 min under the assay condition. *Bacillus amyloliquefaciens* amylase was used as a positive control for the test.

For amylase assay, 1 mL of 1 % (w/v) starch solution in 20 M sodium phosphate buffer at pH 7.0 was pre-equilibrated to 55 °C in a water bath. 1 mL of enzyme solution appropriately diluted in ultra-pure water is added and swirled gently. The mixture was incubated exactly for 3 min. DNS color regent (1 mL) is added immediately and covered with a vented cap. The solution was boiled for exactly 15 min and cooled to room temperature in ice bath. Ultra-pure water (9 mL) is added, mixed by inversion and absorbance reading was taken at 540 nm against the blank solution without the enzyme.

Standard maltose solution was prepared by adding 0.05, 0.2, 0.4, 0.6, 0.8, 1.0 and 2.0 mL of 0.2 %(w/v) maltose standard in a tube. Ultra-pure water was added to the final volume of 2 mL for all standards. A blank solution with 2 mL of water was also prepared. 1 mL of DNS color reagent was added to each tube including blank and boiled in a water bath for exactly 15 min. The solution was cooled to room temperature in ice bath and 9 mL of water is added. Absorbance was taken at 540 nm against the blank. Maltose released (mg) in the amylase test solution was calculated by linear regression analysis of the standard maltose curve. Protein concentration in the enzyme sample was measured by Lowry’s assay [22].

Units/mL enzyme = (mg of maltose released) * Dilution factor

Units /mg protein = (Units/mL enzyme) / (mg/mL protein)

### 2.6 Preliminary selection of production medium

The bacteria were cultured in 100 mL volume in predefined five different basal medium **M1** [23], **M2**, **M3** [24], **M4** [25] and **M5** [23] and incubated at 55 °C. Medium **M1** had the following composition(grams per liter) : Corn starch, 15.0; CaCl_2_H_2_O, 1.0; MgCl_2_.6H_2_O, 1.0; K_2_HPO_4_, 4.0; (NH_4_)_2_SO_4_, 1.0; and trace metal solution 10 mL of composition(milligram per liter) : CuSO_4_.5H_2_O, 16.0; FeSO_4_.7H_2_O, 100.0; ZnSO_4_.7H_2_O, 80.0; MnCl_2_.4H_2_O, 7.0; pH adjusted to 7.0 after autoclaving. Medium **M2** had the following composition (gram per liter): Corn starch, 10.0; Yeast extract, 3.0; Peptone, 5.0; CaCl_2_.2H_2_O, 0.25.0. pH adjusted to 7.0 after autoclaving. Medium **M3** had the following composition (gram per liter): Casein Hydrolysate, 5.0; Yeast Extract, 0.5; K_2_HPO_4_, 3.0; KH_2_PO_4_, 1.0; Soluble starch, 1.0; Trace metal 10 mL of the composition (grams per liter):FeCl_3_, 0.3; MgCl_2_.6H_2_O, 0.5; CaCl_2_.6H_2_O, 0.85; NH_4_Cl, 100.0; NaCl, 100.0. pH adjusted to 7.3 after autoclaving. Medium **M4** had the following composition (grams per liter): Soluble starch, 10.0; Peptone, 5.0; Yeast extract, 2.0; NaCl, 15.0; KH_2_PO_4_, 0.5; MgSO_4_.7H_2_O, 0.5; CaCl_2_, 0.1; Sterile glycerol, 15 % V/V. Medium **M5** had the following composition (grams per liter) : Bacterial peptone, 5.0, Corn starch, 20.0; K_2_HPO_4_, 2.0; (NH_4_)_2_SO_4_, 5.0; MgSO_4_.7H_2_O, 1.0; Sodium lactate, 8.0; CaCO_3_, 2.0; Inositol, 0.01; Trace metals: same as in medium 1. pH was adjusted to 7.0. The cell free medium after centrifugation was tested for amylolytic activity as explained in Section 2.5. The medium with highest amylolytic activity was chosen as basal medium for enzyme production in 300 mL medium.

### 2.7 Purification of amylase

The production medium was centrifuged at 6000 rpm for 15 min to separate cells and particulate matters. The supernatant is collected. Ammonium sulphate was added to the culture supernatant to get 75 % saturation level. All precipitation work was performed at 4 °C. The precipitated solution was centrifuged at 15,000 rpm for 15 min at 4 °C. The pellet was dissolved in 1 mL of 0.01 mM phosphate buffer, pH 7.0. Overnight dialysis was performed using 12,000 MW cut-off dialysis bags against 0.01 M phosphate buffer with three changes of the same buffer and dialysate is centrifuged at 15,000 rpm at 4 °C. The dialyzed solution was purified in a DEAE-Cellulose mini-column. The column was washed with 0.01 M phosphate buffer. Enzyme solution was added slowly and again washed with the same buffer. The enzyme was eluted with 1 M NaCl with a flow rate of 1 mL per min and fractions of 1 mL each was collected. All the fractions were analyzed for amylase assay by DNS assay at 55 °C and pH 7.0. Three fractions with the highest amylase activity were collected and dialyzed against 0.1 M phosphate buffer overnight and freeze-dried (Telstar Lyoquest). Denaturing SDS-PAGE of the purified enzyme was performed according to Laemmli [26] for determination of molecular weight and purity of the enzyme. The gel image was analyzed by GelAnalyzer 19.1.

### 2.8 Study of enzyme optimization, thermostability and enzyme kinetics

#### 2.8.1 Temperature optimization

The effect of temperature on amylase activity was measured from 30 °C to 80 °C at pH 7.0. The amylase activity of appropriately diluted stock enzyme solution was measured as mentioned in section 2.5 but the incubation temperature was varied accordingly. The optimum temperature was determined from the plot of temperature verses relative activity. Relative activity was calculated by taking percentage ratio against the highest activity observed.

#### 2.8.2 pH optimization

The effect of pH on the alpha-amylase activity was performed in the pH range of 3.0−9.0. The activity of diluted stock enzymes was measured as explained in Section 2.5. The starch solution was prepared in different pH adjusted buffers. The buffers used were: 20 mM Sodium acetate (pH 3.0–5.0), 20 mM Sodium phosphate (pH 5.0–7.5) and 20 mM Tris−HCl buffer (pH 8.0–9.5).

#### 2.8.3 Thermostability study

The thermostability of the enzyme was studied at 80 °C. 10 mL of the appropriately diluted enzyme solution was transferred in test tubes and incubated in a water bath at 80 °C at pH 7.0. The residual activity of the enzyme was calculated after 1, 2, 3 and 4 h of incubation at pH 7.0 and 55 °C. Enzyme activity is compared with the activity at time 0 h. as 100 %. The data was fit into the exponential model using Minitab19.0 to calculate half-life.

The kinetic parameter V_max_ and K_m_ was determined by incubating the amylase with the final concentration of soluble starch ranging from 0.1 % to 0.7 % in the buffer at optimum pH and temperature. The enzyme activity was measured as explained in section 2.5. The kinetic parameter V_max_ and K_m_ is determined by nonlinear regression statistical methods using Minitab 19.

### 2.9 Thin layer chromatography

TLC of the hydrolysis product is performed according to Hansen, [27]. The product liberated by the action of amylase on starch is identified by spotting the starch digest and standard sugars (glucose, maltose and maltotrioses) on a pre-coated silica plate activated at 110 °C. The plates were developed in butanol: ethanol: water solvent (5:3:2) and dried overnight at room temperature. The individual sugars were visualized with aniline-diphenylamine reagent.

### 2.10 Enzyme inhibition by metals ions

The stock enzyme solution was added to an equal volume of 2X metal solutions to make a final concentration of 0.1 mM, 1 mM and 10 mM respectively. The salt solution used were BaCl_2_, CaCl_2_, CoCl_2_, CuSO_4_, FeSO_4_, HgCl_2_, KCl, MgSO_4_, MnSo4, NiCl_2_, Pb(C2H3O2)_2_, SnCl_2_ and ZnSO_4_. The activity of the diluted enzyme was measured at optimum temperature and pH.

### 2.11 In situ liquefaction of algal biomass

Alpha-amylase from *A. pallidus* BTPS-2 was tested for the liquefaction efficiency of the algal biomass. The dried biomass of *Chlorella vulgaris* was provided by Algal Research Laboratory, Department of Biotechnology Kathmandu University. The starch content in algal biomass was quantified by Megazyme starch assay kit K-TSTA-100A. 50 mg/mL of dried algal sample was prepared in 0.01 M phosphate buffer at pH 7.0. The algal sample was sonicated at 20 kHz, maximum intensity in Biologics 150 V T ultrasonic Homogenizer to disrupt algal cells. The sonicated sample was boiled at 100 °C water bath for 10 min to gelatinize the starch. The treated biomass was aliquoted in the Gilmont GV2200 viscometer ensuring no leakage. The sample was allowed to equilibrate to 70 °C in a water bath. The units/mL of the enzyme from *A. pallidus* BTPS-2 and *Bacillus amyloliquefaciens* were measured at pH 7.0 and 70 °C. 50 units of the enzyme was added to each algal sample. An algal sample without enzymes was taken as control. The viscosity of the samples was quantified by measuring the time of decent of the glass ball in the interval of 10 min for 1 h. Each sample was run in triplicates. The difference in means was measured by one-way ANOVA at 95 % confidence interval and the means were compared by Tukey’s mean comparison method. The relative viscosity reduction percentage of all samples was measured against the viscosity of the control.

## 3 Results

### 3.1 Sample collection and isolation

Water samples, algal bio-mats and sediments were collected from Bhurung geothermal springs of Nepal. The maximum temperature of the water sample was 72 °C and pH 7.0. A total of 15 strains were isolated based on colony morphology in nutrient agar plates. Only one isolate (BTPS2) showed significant amylase activity in starch agar plates assay.

### 3.2 Characterization

#### 3.2.1 Molecular characterization

**Fig. 1:**
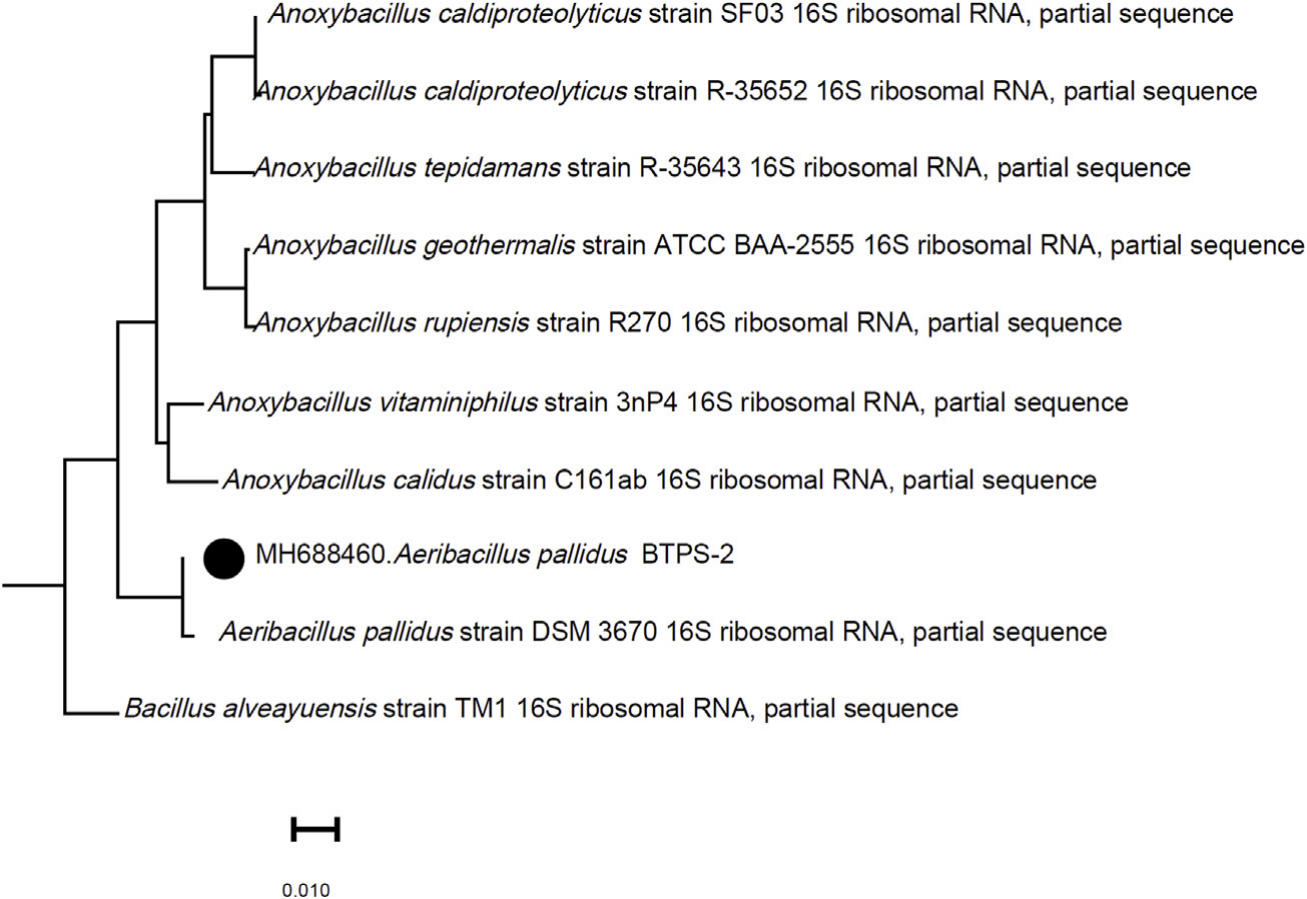
Phylogenetic tree of BTPS2 strain with 99.8 % homology with *Aeribacillus pallidus* DSM 3670 type strain. The tree was constructed using neighbor joining algorithm in rRNA database using NCBI blast tool.

The Blast analysis of 16 s partial sequence of BTPS-2 strain showed 99.8 % homology with the type strain *A. pallidus* DSM 3670. The sequence was published in NCBI gene database with the gene accession no. MH688460. The phylogenetic tree was constructed using the Neighbor-Joining algorithm using NCBI Blast tool (Fig. 1).

#### 3.2.2 Study of morphological, physiological and biochemical properties

**Fig. 2:**
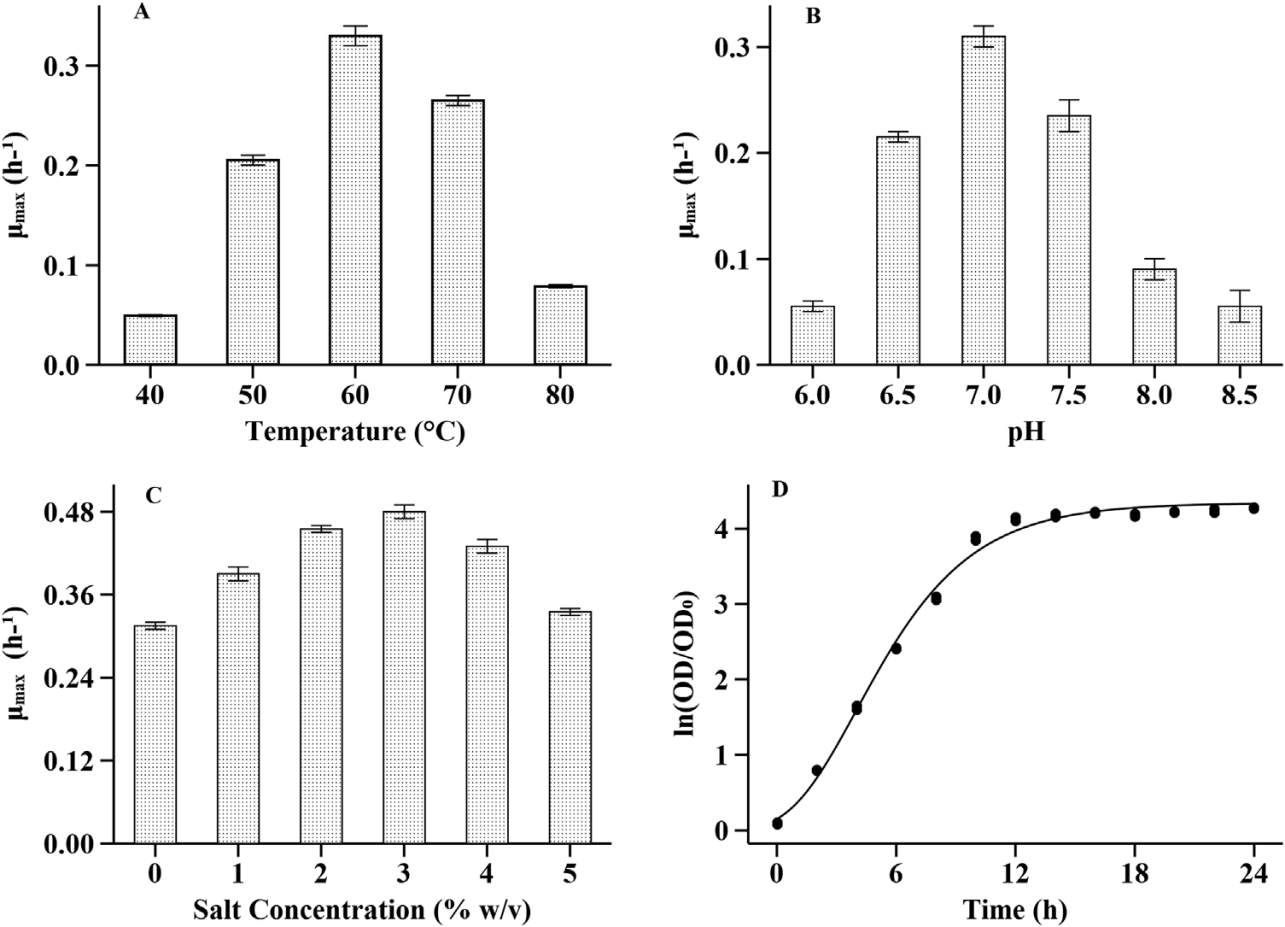
Growth parameter optimization for *A. pallidus* BTPS-2 in nutrient broth. **A**: Maximum specific growth rate(μ_max_) at different temperature. **B**: Maximum specific growth rate (μ_max_) at 60 °C and different pH. **C**: Maximum specific growth rate (μ_max_) at pH 7.0, temperature 60 °C and different salt concentrations. **D**: Gompertz plot of ln (OD/OD_0_) at different time interval; OD_0_: initial optical cell density in McFarland Units.

*A. pallidus* BTPS-2 was gram-positive motile rods with characteristics similar to type strain *A. pallidus* DSM 3670. Morphological, physiological and biochemical properties of *A. pallidus* BTPS-2 are shown in Table 1. The optimal temperature, pH and salt concentration were found to be 60 °C, 7.0 and 3.0 % respectively. The generation time at optimal growth condition was 1.82 h (Fig. 2).

**Table 1:** Taxonomical Characterization:

Test	Study	Results
Morphology	Microscopic study	Gram positive, rods, subterminal endospores
	Colony morphology (48 h incubation on Nutrient broth, 550C)	Circular, pale yellow color, Entire/lobed margin, Flat elevation, Butyrous texture, Opaque, 3−4 mm diameter

Physiological test	Growth on Nutrient broth	Optimum temperature 60 °C, Optimum pH 7.0, Optimum salt tolerance 2.5 %
	Motility in SIM agar	Motile
	Other physiological tests	-ve: Voges-Proskauer test), Gas from fermentation (TSI), Phenylalanine deaminase, anaerobic growth
		+ve: Nitrate reduction, catalase, oxidase

Biochemical Test	Substrate hydrolysis	+ve Starch, Tributyrin, Cellulose, Esculin
		-ve Urea, Gelatin, Casein.
	Acids from Sugar	+ve: Maltose, Fructose, Dextrose, Raffinose, Trehaloes, Melibiose, Sucrose, l-arabinose, Mannose
		-ve: Lactose, Xylose
	Utilization of Carbohydrates and its derivatives	+ve: Glycerol, Salicin, Dulcitol, Inositol, Sorbitol, Mannitol, Arabitol, Alpha-Methyl-d-glycoside, Rhamnose, Cellobiose, Alpha-Methyl-D mannoside, Esculin, Sorbitol, Pyruvate
		-ve: Inulin, Sodium gluconate, Adonitol, Erythritol, Melezitose, Xylitol, ONPG, d-arabinose, Citrate, Malonate, acetate
	Antibiotic sensitivity assay	Sensitive: Carbenicillin, Ciprofloxacin, Gentamicin, Cefotaxime, Cefuroxime, Moxifloxacin, Levofloxacin, Erythromycin, Vancomycin, Rifampicin, Amoxicillin
		Resistant: Ampicillin, Penicillin, Tetracycline, Miconazole, Azithromycin

Molecular Characterization	16 s sequence analysis (PCR Primers: 27 F, 1492R)	99.8 % similarity with *Aeribacillus pallidus* DSMZ 3670

### 3.3 Medium selection and purification

**Fig. 3:**
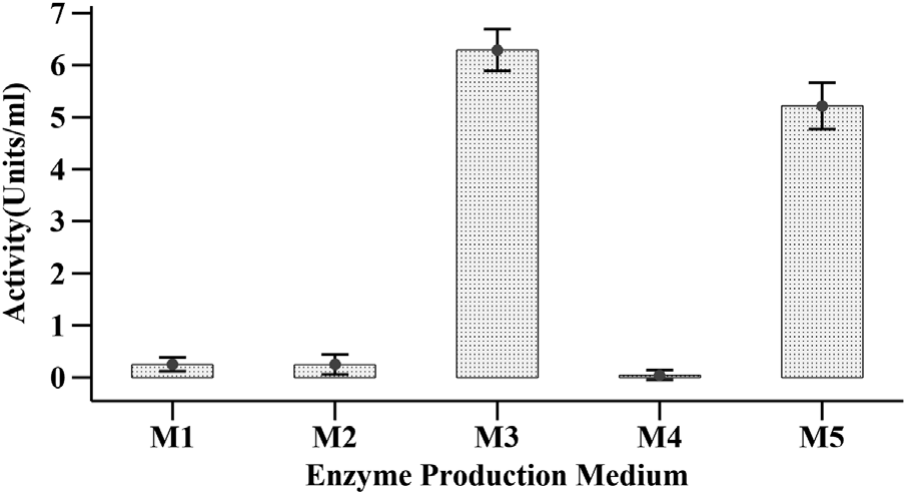
Amylase activity of medium after 48 h incubation.

**Fig. 4:**
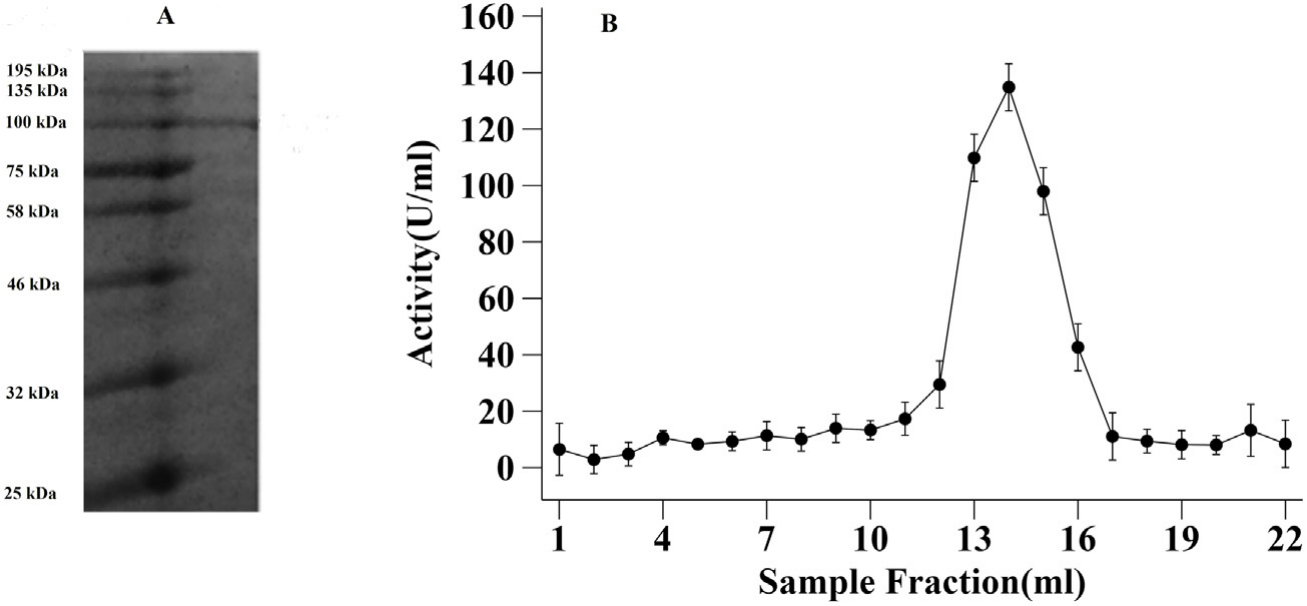
Elution profile and SDS-PAGE analysis. **A**: SDS-PAGE of purified alpha amylase from A. pallidus BTPS-2; Lane 1: Molecular marker, Lane 2: protein band **B:** Enzyme activity of different fraction after DEAE-Cellulose anion exchange chromatography.

The maximum amylase activity was observed in Medium **M3** (6.29 ± 0.18 Units/mL) and medium **M5** (5.22 ± 0.2 Units/mL) while the enzyme production in medium **M1, M2** and **M4** were insignificant (Fig. 3). The enzyme from medium **M3** was purified to 19-fold purification by DEAE-cellulose anion exchange chromatography. The elution pattern showed major peaks of amylase activity for three fractions which were collected (Fig. 4A). The purified fraction was further subjected to SDS-PAGE analysis for the determination of molecular weight. A single peak of the enzyme at 100 kDa was obtained (Fig. 4B). The specific activity of the purified enzyme was 149.6 U/mg protein. The final yield was 38.87 % (Table. 2).

**Table 2:** Purification of alpha-amylase from *Aeribacillus pallidus* BTPS-2.

Steps	Volume (ml)	Activity (U)±SE	Total Protein (mg)±SE	Specific Activity (U/mg) ±SE	Purification Fold	Yield (%)
Broth Supernatant	200	872.90 ± 1.06	111.96 ± 0.3	7.80 ± 0.02	1	100
Precipitation	1.2	462.97 ± 0.71	17.93 ± 0.06	25.82 ± 0.05	3.31	53.04
DEAE cellulose Chromatography	3	339.29 ± 0.83	2.27 ± 0.02	149.60 ± 0.42	19.19	38.87

### 3.4 Enzyme optimization, thermostability and enzyme kinetics

**Fig. 5:**
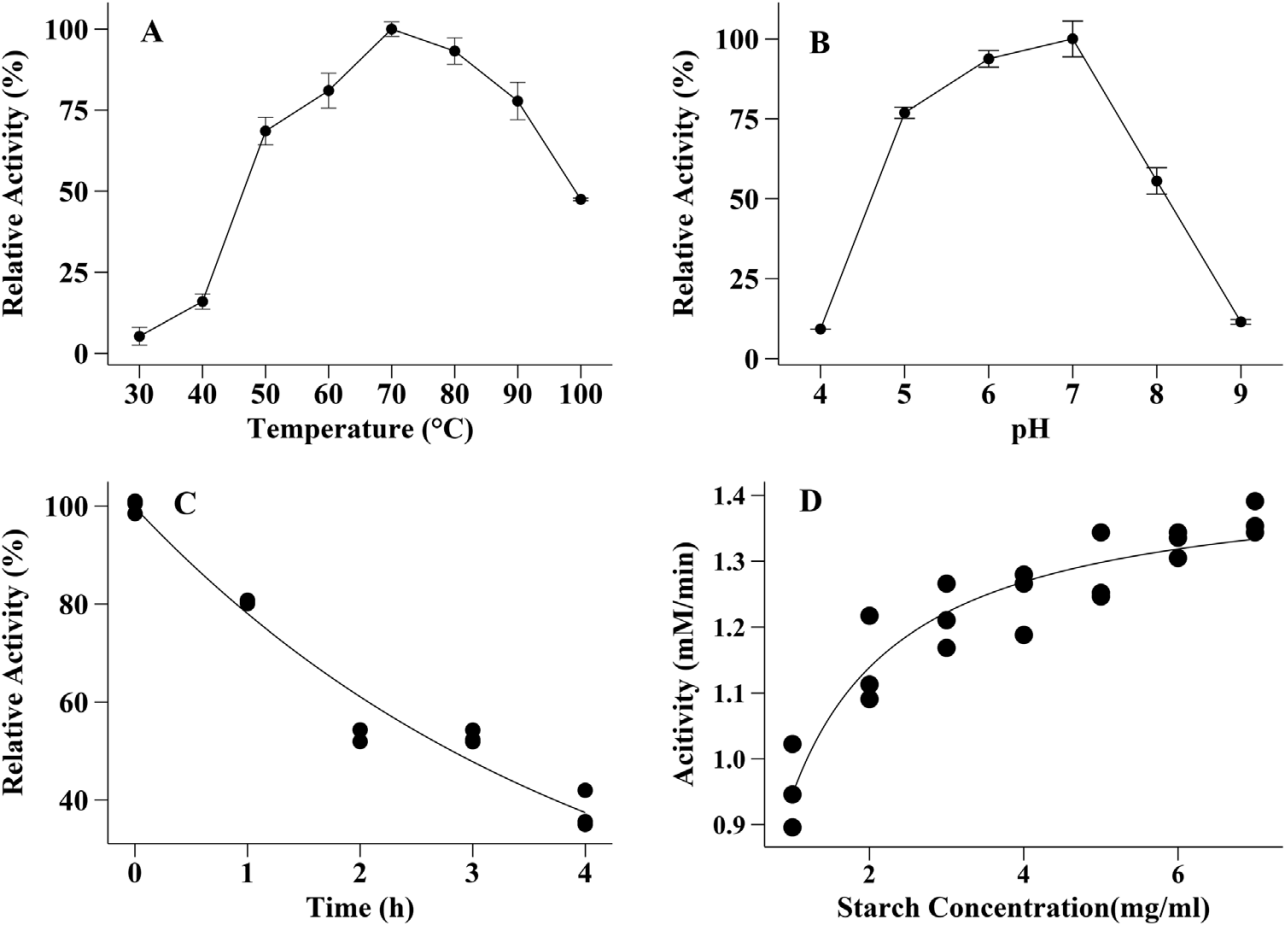
Temperature and pH optimization, Thermostability and Determination of Kinetic parameter. **A**: Effect of pH on Enzyme activity of *A. pallidus* BTPS-2. 100 % activity of amylase = 1.30 U/mL. **B**: Effect of temperature on the enzyme activity of alpha-amylase from *A. pallidus* BTPS-2 at pH 7.0. 100 % activity of amylase = 1.14 U/mL, **C**: Study of thermal stability of alpha amylase from *A. pallidus-*BTPS-2. Enzyme activity at 0 h was considered as 100 %. The half-life of the enzyme was calculated to be 2.81 h. **D**: Kinetics of alpha amylase from *A. pallidus* BTPS-2. The data was fit into the Michaelis-Menten equation using the nonlinear regression method. Estimated K_m_ = 0.51 mg/mL.

The optimum temperature of the enzyme was 70 °C. However, the enzyme retained 93 % activity at 80 °C and 81 % at 60 °C and 47 % at 100 °C as shown in Figure **5A.** The data suggest the thermophilic nature of the enzyme. The optimum pH was found to be at pH 7.0 (Fig. 5B). The enzyme had activity higher than 84 % in the pH range of 6.0–8.0. The thermal stability of the enzyme at 80 °C was studied. The enzyme half-life at 80 °C was 2.82 h (Fig. 5C). The K_m_ and V_max_ values were calculated at optimum temperature and pH using the nonlinear regression method in Minitab 19.0. The Michaelis-Menten constant (K_m_) was 0.51 ± 0.05 mg/mL. The V_max_ value was 1.43 ± 0.02 mM (maltose)/min (Figure. **5D**).

### 3.5 Metal inhibition assay

**Fig. 6:**
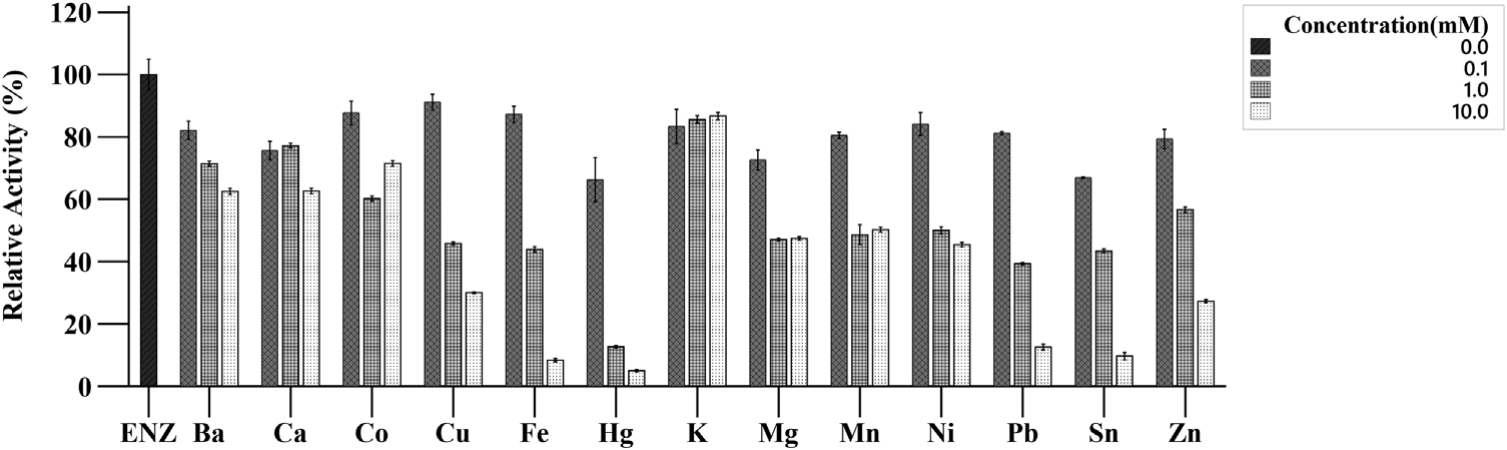
Effect different metal ions on enzyme activity. The stock enzyme solution was mixed with the salt solution to make final concentration of 0.1 mM, 1 mM and 10 mM at pH 6.0. The activity of enzyme treatment without salt is standardized to 100 % activity.

Alpha-amylase activity at 0.1 mM, 1 mM and 10 mM was studied (Fig. 6). All metal ions except K^+^ showed inhibitory effect in the concentration range of 0.1 mM–10 mM. The relative activity of all metal ions investigated were >60 % in 0.1 mM concentration; the maximum inhibition was observed for Hg^+^ ions with relative activity of 68 %. A stronger inhibitory effect was observed in the presence of Fe^2+^, Pb^2+^, Sn^2+^ and Hg^2+^ at 10 mM concentration.

### 3.6 Thin layer chromatography

**Fig. 7:**
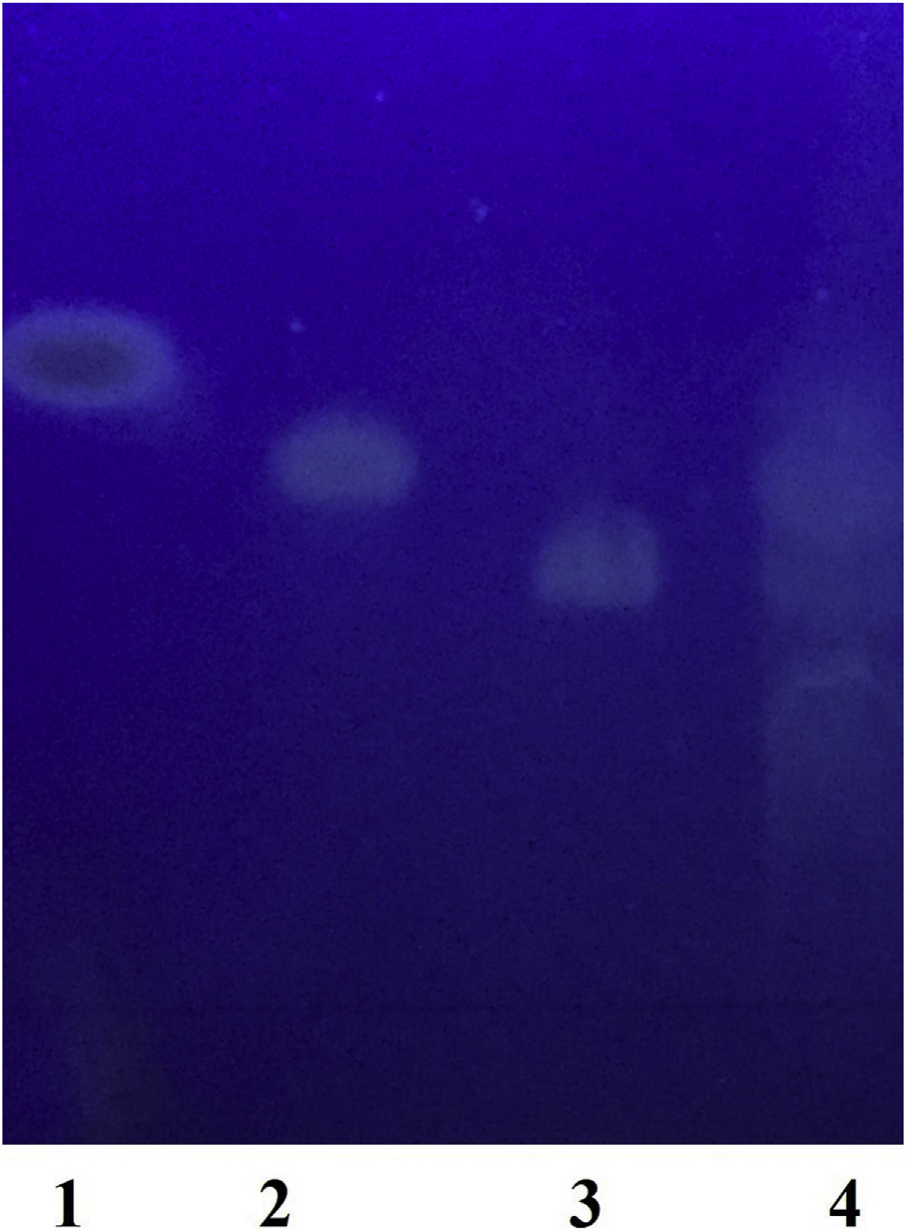
TLC of the hydrolysis product of alpha-amylase from *Aeribacillus pallidus* BTPS-2 with soluble potato starch as substrate. Lane 1: d-glucose, 2: maltose, 3: maltotriose, 4: hydrolysis product of *A. pallidus* BTPS-2 alpha-amylase after 1 h incubation.

To characterize the mode of action of the alpha-amylase, the hydrolysis products were analyzed by thin-layer chromatography. Soluble starch was converted into glucose maltose and maltotriose (Fig. 7). After 1 h incubation, all hydrolysis product was present (Fig. 7**)**. The dominant product was maltose and maltotriose seen as distinct spots in the TLC plate. A smear of band corresponding to oligosaccharide with 6–8 degree of polymerization is visible. This suggests that the alpha-amylase of *A. pallidus* BTPS-2 is a liquefying alpha-amylase.

### 3.7 Enzymatic liquefaction of algal biomass

**Fig. 8:**
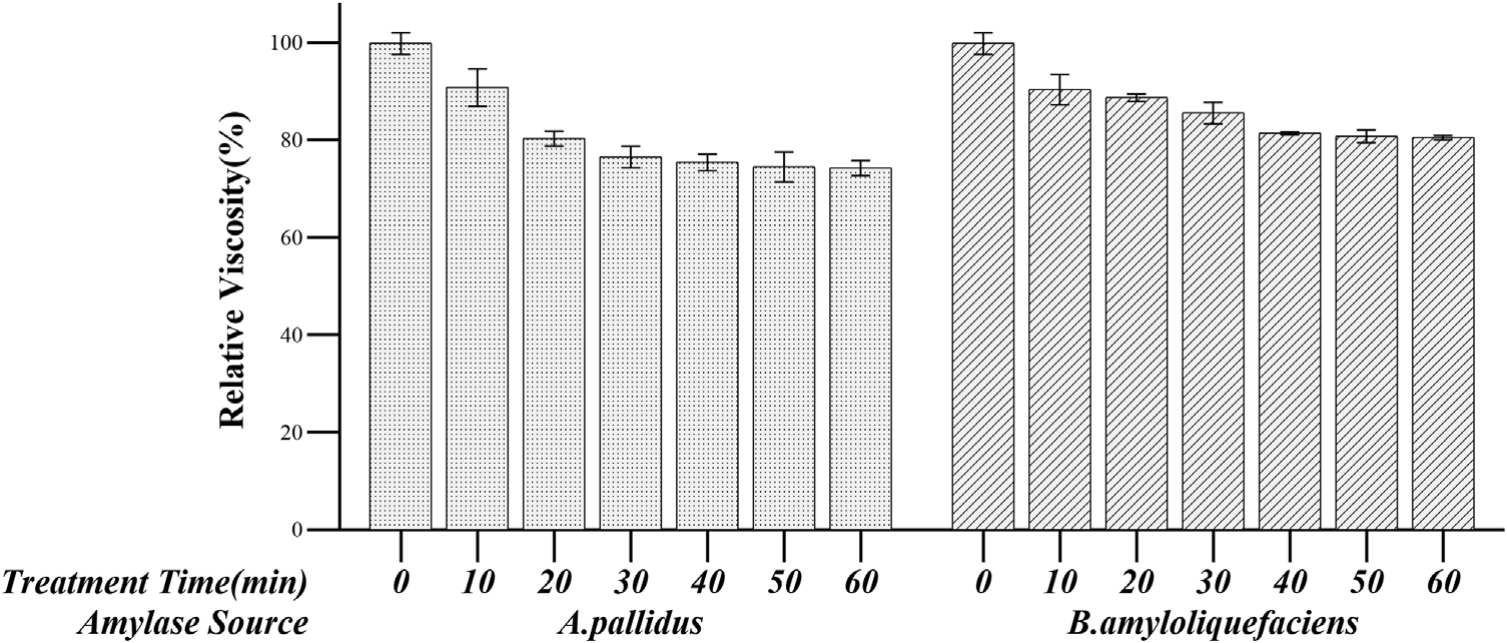
Relative viscosity of enzyme treated algal biomass. 100 % viscosity = 7.35 cp.

Liquefaction of algal biomass by *A. pallidus* BTPS-2 amylase was compared with *B.amyloliquefaciens* (Fig. 8). The starch content in dried *chlorella vulgaris* biomass was estimated to be 27.1 ± 0.38 %(w/w). The sample treated with alpha-amylase from *A. pallidus* BTPS-2 showed viscosity reduction up to 74.2 % after 60 min incubation. The viscosity reduction ratio of alpha-amylase from *B. amyloliquefaciens* was 80.5 %.

## 4 Discussion

The morphological physiological and biochemical properties of *A. pallidus* BTPS-2 were compared with the properties of *A. pallidus* DSM3670 as reported by Scholz et al. [10]. Both are gram-positive, sporulating rods. The colony was circular pale-yellow flat and opaque after 24 h incubation in nutrient broth. Both strains have an optimum temperature of 60 °C. *A. pallidus* BTPS-2 was neutrophilic with a pH optimum of 7.0 in comparison to *A. pallidus* DSM 3670 with pH optimum of 8.0. Both are obligate aerobes and motile. Both were positive for catalase and oxidase test. Hydrolysis of starch, esculin and tributyrin were positive for both strains. Similarly, hydrolysis of casein and gelatin was negative for both strains. Both strains showed similar results in sugar utilization tests. Both strains were negative for lactose, l-arabinose, d-arabinose, trehalose, sorbitol, xylose and malic acid. Utilization of urea, citrate and nitrate reduction test were negative for both strains. BLAST analysis showed that the 16 s rRNA sequence of *A. pallidus* BTPS-2 was 99.8 % similar with the type strain. The morphological, physiological, biochemical and molecular data showed that the isolated bacterial culture is a new strain of *A. pallidus*.

*A. pallidus* BTPS-2 showed strong amylolytic activity in the starch agar plate. Preliminary screening was carried out in five different medium formulations to identify the optimum medium for amylase production. Higher amylase activity in the fermentation broth was observed in medium **M3** and **M4**. Medium **M3** and **M4** both contain complex source of nitrogen as well as trace metal salt supplement (yeast extract and casein hydrolysate in **M3** and peptone in **M5**). The incorporation of complex mediums such as peptone and yeast extract along with the addition of trace metal increased the amylase production by several folds. *A. pallidus* are fastidious halophilic microbes requiring complex salt-rich medium for their growth. This is also reflected by the result that the strains were negative for casein hydrolysis. Their optimum salt concentration was 3 % which reflects their halophilic property. As alpha-amylase is a primary metabolite, the incorporation of casein hydrolysates or yeast extract along with salt solution may have provided optimum growth condition which resulted in higher amylase production.

SDS-PAGE result showed that the molecular mass of *A. pallidus* BTPS-2 alpha-amylase was 100 kDa. This is the first report of the experimental calculation of the molecular mass of *A. pallidus*. However, amylase from other closely related *Geobacillus* species has been reported. The molecular mass of *Geobacillus s*p. IIPTn alpha-amylase was 97 kDa [28]. High molecular mass amylase has also been reported in some strains of *Geobacillus stearothermophilus* [29].

The half-life of *A. pallidus* BTPS-2 alpha-amylase at 80 °C was 2.81 h. The half-life of other bacterial alpha-amylases has been reported. The enzyme from *Bacillus sp*. displayed a half-life of 48 min at 80 °C [30]. The half-life of *B. licheniformis* 44MB82 was 10 min at 85 °C which increased to 120 min in 5 mM CaCl_2_. [31]. Alpha-amylase with higher temperature stability have been reported from *Bacillus amyloliquefaciens* which retained 100 % activity for 24 h at 65 °C and exhibited a half-life of 9 h at 80 °C [32]. The half‐life value of 5 h at 90 °C is reported for starch hydrolyzing alpha‐amylase of *Anoxybacillus rupiensis* TS‐4 [33]. In comparison to previously reported half-life estimates, the alpha-amylase of *A. pallidus* BTPS-2 is moderately thermostable at 80 °C.

The K_m_ value of the alpha-amylase on starch was calculated to be 0.51 mg /mL for soluble potato starch as a substrate. This is lower than other reported value for commercially important amylase source (*Bacillus licheniformis*, K_m_: 0.9 mg/mL [31], *Bacillus amyloliquefaciens* BH072, K_m_: 4.27 mg/mL [34] and *Bacillus subtilis*, K_m_:2.68 mg /mL [35]. Lower K_m_ value reflects the higher affinity of alpha-amylase of *A. pallidus* BTPS-2 for soluble potato starch.

All metal salts investigated except Hg^+^ ions did not show significant inhibition in 0.1 M concentration. The maximum inhibition in 0.1 mM was found for Hg^+^ ions at 68 %. Alpha-amylases with no obligate requirements for metal ions have been reported in other bacterial species [36]. Most of the alpha-amylases are calcium-dependent and exhibit poor performance in the simultaneous saccharification and fermentation process that uses starch as feedstock [37]. The problem can be overcome by using salt tolerant alpha-amylase. Owing to its tolerance to different metal ions in the concentration range of 0.1 mM–1 mM, alpha-amylase from *A. pallidus* BTPS-2 could be an important enzyme for industrial applications.

The study showed that the alpha-amylase from *A. pallidus*-BTPS2 has more liquefying efficiency for algal biomass compared to alpha-amylase from *Bacillus amyloliquefaciens*. Microalgae are a good absorber of heavy metals from solution [38]. The enzyme activity can be affected by these heavy metals during in situ saccharification process. In our study, the increased efficiency of alpha-amylase from *A. pallidus* BTPS-2 could be due to its specificity for algal starch as well as the presence of various metal ions in the algal broth.

## 5 Conclusion

This is the first report of purification and characterization of alpha-amylase from *Aeribacillus pallidus* strain. The liquefying maltogenic alpha-amylase was found to be highly thermostable and insensitive to metal inhibition. The enzyme showed higher liquefying efficiency for algal starch than alpha-amylase from *B. amyloliquefaciens*. The enzyme could be a potential candidate for industrial application.

## CRediT authorship contribution statement

**Parash Mani Timilsina:** Conceptualization, Methodology, Visualization, Writing - original draft, Software, Validation, Funding acquisition, Data curation, Investigation, Writing - review & editing. **Gyanu Raj Pandey:** Investigation. **Asmita Shrestha:** Investigation. **Manish Ojha:** Investigation. **Tika Bahadur Karki:** Data curation, Supervision, Writing - review & editing.

## Declaration of Competing Interest

The authors report no declarations of interest.
